# 5-(4-Chloro­phen­yl)-1-methyl-3-phenyl-3,6,8,9-tetra­hydro­pyrazolo­[3,4-*b*]thio­pyrano[4,3-*d*]pyridine

**DOI:** 10.1107/S1600536811033514

**Published:** 2011-08-27

**Authors:** Runhong Jia, Juhua Peng

**Affiliations:** a Lianyungang Teacher’s College, Lianyungang 222006, People’s Republic of China

## Abstract

The title compound, C_22_H_18_ClN_3_S, was synthesized by the reaction of 4-chloro­benzaldehyde, tetra­hydro­thio­pyran-4-one and 3-methyl-1-phenyl-1*H*-pyrazol-5-amine in acetic acid without a catalyst. The pyridine and pyrazole rings are almost coplanar, the dihedral angle between their mean planes being 2.50 (1)°. The thio­pyran ring exhibits an envelope conformation. The crystal packing is stabilized by inter­molecular C—H⋯Cl hydrogen bonds and by C—H⋯π and π–π inter­actions [centroid–centroid distances of 3.825 (2) Å between pyridine rings and 3.557 (2) Å between pyrazole and pyridine rings.

## Related literature

The pyrazolo­[3,4-*b*]pyridine system represents the core skeleton of a pharmaceutically important class of heterocyclic compounds that possess a broad range of biological activity, see: Beutner *et al.* (2009 ▶); Hamblin *et al.* (2008 ▶); Jiang *et al.* (2011 ▶); Lynck *et al.* (1988 ▶); Manetti *et al.* (2005 ▶); Meiners & Salama (1982 ▶); Revesz *et al.* (2006 ▶); Witherington *et al.* (2003 ▶). For related structures, see: Chebanov *et al.* (2007 ▶); Lee & Park (2009 ▶); Quiroga *et al.* (2001 ▶).
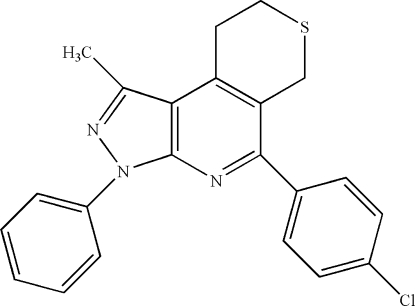


## Experimental

### 

#### Crystal data

C_22_H_18_ClN_3_S
*M* *_r_* = 391.90
Monoclinic, 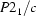

*a* = 8.8731 (9) Å
*b* = 19.9044 (18) Å
*c* = 10.5292 (11) Å
β = 96.689 (1)°
*V* = 1846.9 (3) Å^3^
*Z* = 4
Mo *K*α radiation
μ = 0.33 mm^−1^
*T* = 298 K
0.48 × 0.19 × 0.18 mm

#### Data collection

Bruker SMART CCD area-detector diffractometer
Absorption correction: multi-scan (*SADABS*; Sheldrick, 1996) ▶ *T*_min_ = 0.857, *T*_max_ = 0.943
9193 measured reflections
3264 independent reflections
1988 reflections with *I* > 2σ(*I*)
*R*_int_ = 0.049

#### Refinement

*R*[*F* ^2^ > 2σ(*F* ^2^)] = 0.046
*wR*(*F* ^2^) = 0.118
*S* = 1.02
3264 reflections
245 parameters
H-atom parameters constrained
Δρ_max_ = 0.24 e Å^−3^
Δρ_min_ = −0.32 e Å^−3^

### 

Data collection: *SMART* (Bruker, 1998 ▶); cell refinement: *SAINT* (Bruker, 1999 ▶); data reduction: *SAINT*; program(s) used to solve structure: *SHELXS97* (Sheldrick, 2008 ▶); program(s) used to refine structure: *SHELXL97* (Sheldrick, 2008 ▶); molecular graphics: *SHELXTL* (Sheldrick, 2008 ▶); software used to prepare material for publication: *SHELXTL*.

## Supplementary Material

Crystal structure: contains datablock(s) global, I. DOI: 10.1107/S1600536811033514/zq2115sup1.cif

Structure factors: contains datablock(s) I. DOI: 10.1107/S1600536811033514/zq2115Isup2.hkl

Supplementary material file. DOI: 10.1107/S1600536811033514/zq2115Isup3.cml

Additional supplementary materials:  crystallographic information; 3D view; checkCIF report

## Figures and Tables

**Table 1:** Hydrogen-bond geometry (Å, °) Please define *Cg*1 is the centroid of the C17–C22 ring.

*D*—H⋯*A*	*D*—H	H⋯*A*	*D*⋯*A*	*D*—H⋯*A*
C5—H5*A*⋯Cl1^i^	0.97	3.00	3.608 (3)	122
C9—H9*A*⋯*Cg*1^ii^	0.97	2.84	3.778 (4)	163

Symmetry codes: (i) 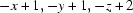; (ii) 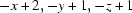.

**Fig. 1.**
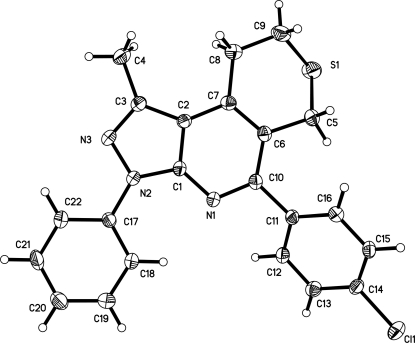
The molecular structure of the title compound showing 30% probability displacement ellipsoids.

**Fig. 2.**
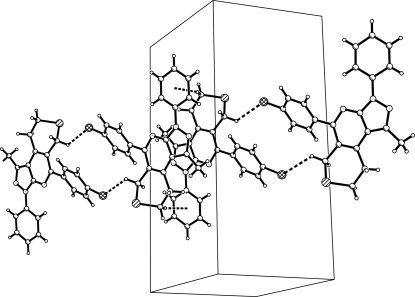
The packing diagram of the title compound viewed along the a axis.

## supplementary crystallographic information

### Comment

The pyrazolo[3,4-*b*]pyridine system as a key heterocycle represents the core skeleton of a pharmaceutically important class of heterocyclic compounds that possess a broad range of biological activities (Beutner *et al.*, 2009), such as anxiolytic activity (Meiners *et al.*, 1982), and can be used in the inhibition of xanthine oxidases (Lynck *et al.*, 1988), cholesterol formation and in the treatment of Alzheimer's disease, gastrointestinal diseases, anorexia nervosa, drug and alcohol withdrawal symptoms, drug addiction and infertility. They have also been reported as potent and selective inhibitors of A1 adenosine receptors (Manetti *et al.*, 2005), phosphodiesterase 4 (PDE4) inhibitors in immune and inflammatory cells (Hamblin *et al.* 2008), glycogen synthase kinase-3 (GSK-3) inhibitors (Witherington *et al.*, 2003) and kinase inhibitors of p38 as anti-inflammatory drugs (Revesz *et al.*, 2006). Because of the biological activities they exhibit, these compounds have distinguished themselves as heterocycles of profound chemical and biological significance.

Thus, the preparation of these molecules has attracted considerable attention (Lee *et al.*, 2009). Many pyrazolo [3,4-*b*]pyridines have been synthesized through the reactions of 5-aminopyrazoles, aldehydes and appropriate cycloketones by various methods (Quiroga *et al.* 2001). However, most of these compounds are pyrazolo[3,4-*b*]pyridines with the aryl group at the 4-position of the pyridine ring. Recently, Chebanov and co-workers synthesized unexpected pyrazolopyridines by similar three-component reactions under strong basic conditions (Chebanov *et al.*, 2007). In this reaction, the aryl group was also located at the 4-position of the pyridine ring. Recently, Jiang *et al.* (Jiang *et al.*, 2011) have reported a facile one-pot reaction for the synthesis of the regioselective construction of macrocyclane-fused pyrazolo[3,4-*b*]pyridines with an aryl group at the 2-position of the pyridine nucleus.

In this paper we report the crystal structure of the title compound, C_22_H_18_ClN_3_S, which was synthesized by the reaction of 4-chlorobenzaldehyde, tetrahydrothiopyran-4-one, and 3-methyl-1-phenyl-1*H*-pyrazol-5-amine in acetic acid without catalyst.

In the crystal structure, the pyridine and pyrazole rings are almost coplanar. Indeed, the dihedral angle between the pyridine C1/C2/C7/C6/C10/N1 plane and the C1/C2/C3/N3/N2 pyrazole ring is 2.50 (1)°. The thiopyran ring exhibits an envelope conformation. The molecules are connected *via* C—H···Cl hydrogen bonds and C—H···π interactions (Fig. 2; Table 1). Furthermore, intermolecular π—π interactions between two parallel neighboring pyridine rings are observed. The centroid-centroid distance and the perpendicular distance of the centroid on the neighboring ring are 3.825 (2) and 3.472 (1). Even shorter interactions exist between the pyrazole and pyridine rings with corresponding distances of 3.557 (2) and 3.516 (1) Å, respectively.

### Experimental

The title compound was prepared by the reaction of 4-chlorobenzaldehyde (1 mmol), tetrahydrothiopyran-4-one (1 mmol) and 3-methyl-1-phenyl-1*H*-pyrazol-5-amine (1 mmol) in acetic acid (2.0 ml). Single crystals suitable for X-ray diffraction were obtained by slow evaporation of a 95% aqueous ethanol solution (yield 89%; m.p. 473–475 K).

IR (cm^-1^): 3068, 2963, 2896, 1592, 1577, 1506, 1415, 1383, 1286, 1104, 1090, 1014, 911, 8839, 756, 693. ^1^H NMR (DMSO-d_6_): 8.25–8.23 (m, 2H, ArH), 7.63–7.61 (m, 4H, ArH), 7.50 (s, 2H, ArH), 7.27 (s, 1H, ArH), 3.78 (s, 2H, CH_2_), 3.61–3.54 (m, 2H, CH_2_), 3.06–3.00 (m, 2H, CH_2_), 2.76 (s, 3H, CH_3_).

### Refinement

All H atoms were positioned geometrically and treated as riding, with C—H = 0.93 Å and *U*_iso_(H) = 1.2*U*_eq_(C) for aromatic H atoms, with C—H = 0.97 Å and *U*_iso_(H) = 1.2*U*_eq_(C) for methylene H atoms, and with C—H = 0.96 Å and *U*_iso_(H) = 1.5*U*_eq_(C) for methyl H atoms.

In the hydrogen-bond geometry table, *Cg*1 corresponds to the centroid of the C17/C18/C19/C20/C21/C22 ring.

### Crystal data

**Table:**

C_22_H_18_ClN_3_S	*F*(000) = 816
*M**_r_* = 391.90	*D*_x_ = 1.409 Mg m^−^^3^
Monoclinic, *P*2_1_/*c*	Melting point = 473–475 K
Hall symbol: -P 2ybc	Mo *K*α radiation, λ = 0.71073 Å
*a* = 8.8731 (9) Å	Cell parameters from 2113 reflections
*b* = 19.9044 (18) Å	θ = 2.2–26.7°
*c* = 10.5292 (11) Å	µ = 0.33 mm^−^^1^
β = 96.689 (1)°	*T* = 298 K
*V* = 1846.9 (3) Å^3^	Solid, yellow
*Z* = 4	0.48 × 0.19 × 0.18 mm

### Data collection

**Table:**

Bruker SMART CCD area-detector diffractometer	3264 independent reflections
Radiation source: fine-focus sealed tube	1988 reflections with *I* > 2σ(*I*)
graphite	*R*_int_ = 0.049
φ and ω scans	θ_max_ = 25.0°, θ_min_ = 2.1°
Absorption correction: multi-scan (*SADABS*; Sheldrick, 1996)	*h* = −10→10
*T*_min_ = 0.857, *T*_max_ = 0.943	*k* = −23→23
9193 measured reflections	*l* = −12→10

### Refinement

**Table:**

Refinement on *F*^2^	Primary atom site location: structure-invariant direct methods
Least-squares matrix: full	Secondary atom site location: difference Fourier map
*R*[*F*^2^ > 2σ(*F*^2^)] = 0.046	Hydrogen site location: inferred from neighbouring sites
*wR*(*F*^2^) = 0.118	H-atom parameters constrained
*S* = 1.02	*w* = 1/[σ^2^(*F*_o_^2^) + (0.0492*P*)^2^ + 0.3375*P*] where *P* = (*F*_o_^2^ + 2*F*_c_^2^)/3
3264 reflections	(Δ/σ)_max_ < 0.001
245 parameters	Δρ_max_ = 0.24 e Å^−^^3^
0 restraints	Δρ_min_ = −0.32 e Å^−^^3^

### Special details

**Table:**

Geometry. All e.s.d.'s (except the e.s.d. in the dihedral angle between two l.s. planes) are estimated using the full covariance matrix. The cell e.s.d.'s are taken into account individually in the estimation of e.s.d.'s in distances, angles and torsion angles; correlations between e.s.d.'s in cell parameters are only used when they are defined by crystal symmetry. An approximate (isotropic) treatment of cell e.s.d.'s is used for estimating e.s.d.'s involving l.s. planes.
Refinement. Refinement of *F*^2^ against ALL reflections. The weighted *R*-factor *wR* and goodness of fit *S* are based on *F*^2^, conventional *R*-factors *R* are based on *F*, with *F* set to zero for negative *F*^2^. The threshold expression of *F*^2^ > σ(*F*^2^) is used only for calculating *R*-factors(gt) *etc*. and is not relevant to the choice of reflections for refinement. *R*-factors based on *F*^2^ are statistically about twice as large as those based on *F*, and *R*- factors based on ALL data will be even larger.

### Fractional atomic coordinates and isotropic or equivalent isotropic displacement parameters (Å^2^)

**Table:**

	*x*	*y*	*z*	*U*_iso_*/*U*_eq_	
Cl1	0.44021 (11)	0.63561 (4)	1.01417 (8)	0.0593 (3)	
N1	0.7485 (3)	0.54523 (11)	0.4998 (2)	0.0332 (6)	
N2	0.8363 (3)	0.55347 (11)	0.2913 (2)	0.0357 (6)	
N3	0.8983 (3)	0.51019 (11)	0.2085 (2)	0.0387 (6)	
S1	0.67456 (11)	0.30052 (4)	0.63061 (9)	0.0578 (3)	
C1	0.8091 (3)	0.51924 (13)	0.4001 (2)	0.0306 (7)	
C2	0.8559 (3)	0.45316 (13)	0.3864 (3)	0.0318 (7)	
C3	0.9106 (3)	0.45062 (14)	0.2642 (3)	0.0369 (7)	
C4	0.9691 (4)	0.39328 (14)	0.1935 (3)	0.0523 (9)	
H4A	0.9980	0.4090	0.1136	0.079*	
H4B	0.8912	0.3598	0.1775	0.079*	
H4C	1.0557	0.3741	0.2436	0.079*	
C5	0.7208 (4)	0.38381 (14)	0.6887 (3)	0.0447 (8)	
H5A	0.6339	0.4023	0.7245	0.054*	
H5B	0.8038	0.3810	0.7571	0.054*	
C6	0.7656 (3)	0.43144 (13)	0.5871 (2)	0.0331 (7)	
C7	0.8341 (3)	0.40722 (13)	0.4839 (3)	0.0328 (7)	
C8	0.8818 (4)	0.33513 (14)	0.4683 (3)	0.0457 (8)	
H8A	0.8294	0.3184	0.3886	0.055*	
H8B	0.9895	0.3348	0.4598	0.055*	
C9	0.8542 (4)	0.28645 (15)	0.5730 (3)	0.0574 (10)	
H9A	0.9344	0.2911	0.6433	0.069*	
H9B	0.8574	0.2409	0.5406	0.069*	
C10	0.7281 (3)	0.50130 (13)	0.5927 (2)	0.0317 (7)	
C11	0.6564 (3)	0.53181 (13)	0.7005 (2)	0.0316 (7)	
C12	0.5455 (3)	0.58021 (14)	0.6731 (3)	0.0368 (7)	
H12	0.5153	0.5916	0.5883	0.044*	
C13	0.4789 (3)	0.61196 (14)	0.7682 (3)	0.0382 (7)	
H13	0.4048	0.6445	0.7480	0.046*	
C14	0.5232 (4)	0.59506 (14)	0.8936 (3)	0.0374 (7)	
C15	0.6343 (4)	0.54796 (14)	0.9245 (3)	0.0392 (8)	
H15	0.6645	0.5372	1.0096	0.047*	
C16	0.7009 (3)	0.51666 (13)	0.8279 (3)	0.0375 (7)	
H16	0.7767	0.4849	0.8487	0.045*	
C17	0.8084 (3)	0.62075 (13)	0.2522 (2)	0.0328 (7)	
C18	0.7261 (3)	0.66428 (14)	0.3209 (3)	0.0406 (8)	
H18	0.6893	0.6498	0.3954	0.049*	
C19	0.6992 (4)	0.72906 (15)	0.2776 (3)	0.0473 (8)	
H19	0.6441	0.7581	0.3238	0.057*	
C20	0.7524 (4)	0.75172 (16)	0.1675 (3)	0.0503 (9)	
H20	0.7331	0.7954	0.1388	0.060*	
C21	0.8344 (4)	0.70828 (15)	0.1010 (3)	0.0489 (9)	
H21	0.8706	0.7230	0.0264	0.059*	
C22	0.8645 (3)	0.64352 (14)	0.1420 (3)	0.0405 (8)	
H22	0.9218	0.6152	0.0963	0.049*	

### Atomic displacement parameters (Å^2^)

**Table:**

	*U*^11^	*U*^22^	*U*^33^	*U*^12^	*U*^13^	*U*^23^
Cl1	0.0792 (7)	0.0579 (6)	0.0456 (5)	0.0061 (5)	0.0279 (5)	−0.0081 (4)
N1	0.0386 (15)	0.0337 (13)	0.0286 (13)	0.0005 (11)	0.0092 (11)	0.0007 (11)
N2	0.0452 (16)	0.0333 (13)	0.0306 (13)	0.0045 (12)	0.0128 (12)	0.0004 (11)
N3	0.0455 (17)	0.0405 (15)	0.0323 (14)	0.0043 (12)	0.0138 (12)	−0.0038 (11)
S1	0.0621 (7)	0.0369 (5)	0.0795 (7)	−0.0095 (4)	0.0298 (5)	0.0017 (4)
C1	0.0312 (17)	0.0326 (16)	0.0290 (15)	−0.0012 (13)	0.0078 (13)	0.0014 (12)
C2	0.0328 (18)	0.0292 (15)	0.0344 (16)	0.0003 (13)	0.0079 (13)	−0.0012 (13)
C3	0.0383 (19)	0.0375 (17)	0.0357 (17)	0.0037 (15)	0.0078 (14)	−0.0023 (14)
C4	0.065 (3)	0.0421 (19)	0.055 (2)	0.0025 (17)	0.0255 (18)	−0.0082 (16)
C5	0.057 (2)	0.0361 (17)	0.0434 (19)	−0.0031 (15)	0.0166 (16)	0.0052 (14)
C6	0.0335 (18)	0.0341 (16)	0.0316 (16)	−0.0033 (13)	0.0037 (13)	0.0019 (12)
C7	0.0310 (18)	0.0292 (16)	0.0380 (17)	−0.0027 (13)	0.0038 (14)	−0.0009 (13)
C8	0.051 (2)	0.0375 (18)	0.0502 (19)	0.0060 (15)	0.0119 (16)	0.0012 (15)
C9	0.066 (3)	0.0341 (18)	0.076 (2)	0.0056 (17)	0.024 (2)	0.0087 (17)
C10	0.0328 (18)	0.0338 (16)	0.0286 (15)	−0.0036 (13)	0.0042 (13)	0.0009 (13)
C11	0.0362 (18)	0.0294 (15)	0.0301 (16)	−0.0054 (13)	0.0082 (13)	0.0003 (12)
C12	0.042 (2)	0.0404 (17)	0.0286 (16)	0.0015 (15)	0.0056 (14)	0.0029 (13)
C13	0.0327 (19)	0.0422 (17)	0.0406 (18)	0.0041 (14)	0.0077 (15)	−0.0011 (14)
C14	0.046 (2)	0.0352 (17)	0.0329 (17)	−0.0074 (15)	0.0150 (15)	−0.0065 (13)
C15	0.053 (2)	0.0380 (17)	0.0281 (16)	−0.0046 (16)	0.0099 (15)	0.0036 (13)
C16	0.043 (2)	0.0341 (16)	0.0364 (17)	0.0026 (14)	0.0080 (15)	0.0028 (13)
C17	0.0382 (19)	0.0301 (15)	0.0303 (16)	−0.0019 (14)	0.0043 (14)	0.0011 (12)
C18	0.052 (2)	0.0391 (18)	0.0326 (17)	0.0015 (16)	0.0128 (15)	0.0020 (14)
C19	0.056 (2)	0.0372 (18)	0.050 (2)	0.0063 (16)	0.0115 (17)	0.0008 (15)
C20	0.056 (2)	0.0395 (19)	0.056 (2)	−0.0026 (17)	0.0088 (18)	0.0091 (16)
C21	0.059 (2)	0.048 (2)	0.0416 (19)	−0.0107 (17)	0.0136 (17)	0.0130 (16)
C22	0.043 (2)	0.0444 (19)	0.0361 (17)	−0.0007 (15)	0.0131 (15)	0.0029 (14)

### Geometric parameters (Å, °)

**Table:**

Cl1—C14	1.739 (3)	C9—H9A	0.9700
N1—C1	1.339 (3)	C9—H9B	0.9700
N1—C10	1.339 (3)	C10—C11	1.493 (4)
N2—C1	1.378 (3)	C11—C12	1.383 (4)
N2—N3	1.384 (3)	C11—C16	1.387 (4)
N2—C17	1.414 (3)	C12—C13	1.374 (4)
N3—C3	1.322 (3)	C12—H12	0.9300
S1—C9	1.792 (3)	C13—C14	1.375 (4)
S1—C5	1.798 (3)	C13—H13	0.9300
C1—C2	1.392 (4)	C14—C15	1.372 (4)
C2—C7	1.405 (4)	C15—C16	1.382 (4)
C2—C3	1.428 (4)	C15—H15	0.9300
C3—C4	1.489 (4)	C16—H16	0.9300
C4—H4A	0.9600	C17—C18	1.389 (4)
C4—H4B	0.9600	C17—C22	1.391 (4)
C4—H4C	0.9600	C18—C19	1.379 (4)
C5—C6	1.517 (4)	C18—H18	0.9300
C5—H5A	0.9700	C19—C20	1.378 (4)
C5—H5B	0.9700	C19—H19	0.9300
C6—C7	1.391 (4)	C20—C21	1.373 (4)
C6—C10	1.433 (4)	C20—H20	0.9300
C7—C8	1.511 (4)	C21—C22	1.375 (4)
C8—C9	1.509 (4)	C21—H21	0.9300
C8—H8A	0.9700	C22—H22	0.9300
C8—H8B	0.9700		
			
C1—N1—C10	114.9 (2)	C8—C9—H9B	109.2
C1—N2—N3	109.7 (2)	S1—C9—H9B	109.2
C1—N2—C17	131.9 (2)	H9A—C9—H9B	107.9
N3—N2—C17	118.4 (2)	N1—C10—C6	123.5 (2)
C3—N3—N2	107.3 (2)	N1—C10—C11	113.3 (2)
C9—S1—C5	94.73 (15)	C6—C10—C11	123.2 (2)
N1—C1—N2	125.9 (2)	C12—C11—C16	117.9 (3)
N1—C1—C2	126.7 (2)	C12—C11—C10	118.7 (2)
N2—C1—C2	107.3 (2)	C16—C11—C10	123.3 (3)
C1—C2—C7	118.1 (2)	C13—C12—C11	121.6 (3)
C1—C2—C3	105.3 (2)	C13—C12—H12	119.2
C7—C2—C3	136.5 (3)	C11—C12—H12	119.2
N3—C3—C2	110.4 (2)	C12—C13—C14	119.2 (3)
N3—C3—C4	118.7 (3)	C12—C13—H13	120.4
C2—C3—C4	130.8 (3)	C14—C13—H13	120.4
C3—C4—H4A	109.5	C15—C14—C13	120.8 (3)
C3—C4—H4B	109.5	C15—C14—Cl1	119.9 (2)
H4A—C4—H4B	109.5	C13—C14—Cl1	119.3 (2)
C3—C4—H4C	109.5	C14—C15—C16	119.4 (3)
H4A—C4—H4C	109.5	C14—C15—H15	120.3
H4B—C4—H4C	109.5	C16—C15—H15	120.3
C6—C5—S1	113.9 (2)	C15—C16—C11	121.0 (3)
C6—C5—H5A	108.8	C15—C16—H16	119.5
S1—C5—H5A	108.8	C11—C16—H16	119.5
C6—C5—H5B	108.8	C18—C17—C22	119.5 (3)
S1—C5—H5B	108.8	C18—C17—N2	121.6 (2)
H5A—C5—H5B	107.7	C22—C17—N2	118.9 (2)
C7—C6—C10	119.6 (2)	C19—C18—C17	119.5 (3)
C7—C6—C5	120.6 (3)	C19—C18—H18	120.2
C10—C6—C5	119.8 (2)	C17—C18—H18	120.2
C6—C7—C2	117.1 (2)	C20—C19—C18	121.4 (3)
C6—C7—C8	124.6 (2)	C20—C19—H19	119.3
C2—C7—C8	118.3 (2)	C18—C19—H19	119.3
C9—C8—C7	117.3 (3)	C21—C20—C19	118.5 (3)
C9—C8—H8A	108.0	C21—C20—H20	120.8
C7—C8—H8A	108.0	C19—C20—H20	120.8
C9—C8—H8B	108.0	C20—C21—C22	121.7 (3)
C7—C8—H8B	108.0	C20—C21—H21	119.2
H8A—C8—H8B	107.2	C22—C21—H21	119.2
C8—C9—S1	111.9 (2)	C21—C22—C17	119.5 (3)
C8—C9—H9A	109.2	C21—C22—H22	120.3
S1—C9—H9A	109.2	C17—C22—H22	120.3
			
C1—N2—N3—C3	−0.4 (3)	C1—N1—C10—C6	−0.8 (4)
C17—N2—N3—C3	−178.7 (2)	C1—N1—C10—C11	−178.4 (2)
C10—N1—C1—N2	177.7 (3)	C7—C6—C10—N1	3.4 (4)
C10—N1—C1—C2	−2.2 (4)	C5—C6—C10—N1	−172.5 (3)
N3—N2—C1—N1	−179.4 (3)	C7—C6—C10—C11	−179.2 (3)
C17—N2—C1—N1	−1.4 (5)	C5—C6—C10—C11	5.0 (4)
N3—N2—C1—C2	0.5 (3)	N1—C10—C11—C12	37.0 (4)
C17—N2—C1—C2	178.4 (3)	C6—C10—C11—C12	−140.7 (3)
N1—C1—C2—C7	2.3 (4)	N1—C10—C11—C16	−139.5 (3)
N2—C1—C2—C7	−177.5 (2)	C6—C10—C11—C16	42.9 (4)
N1—C1—C2—C3	179.5 (3)	C16—C11—C12—C13	−1.0 (4)
N2—C1—C2—C3	−0.4 (3)	C10—C11—C12—C13	−177.6 (3)
N2—N3—C3—C2	0.1 (3)	C11—C12—C13—C14	−0.2 (4)
N2—N3—C3—C4	177.6 (3)	C12—C13—C14—C15	1.1 (4)
C1—C2—C3—N3	0.1 (3)	C12—C13—C14—Cl1	179.5 (2)
C7—C2—C3—N3	176.5 (3)	C13—C14—C15—C16	−0.8 (4)
C1—C2—C3—C4	−176.9 (3)	Cl1—C14—C15—C16	−179.2 (2)
C7—C2—C3—C4	−0.6 (6)	C14—C15—C16—C11	−0.4 (4)
C9—S1—C5—C6	57.4 (3)	C12—C11—C16—C15	1.3 (4)
S1—C5—C6—C7	−29.2 (4)	C10—C11—C16—C15	177.8 (3)
S1—C5—C6—C10	146.6 (2)	C1—N2—C17—C18	−7.6 (5)
C10—C6—C7—C2	−3.1 (4)	N3—N2—C17—C18	170.2 (3)
C5—C6—C7—C2	172.8 (3)	C1—N2—C17—C22	172.7 (3)
C10—C6—C7—C8	178.7 (3)	N3—N2—C17—C22	−9.5 (4)
C5—C6—C7—C8	−5.4 (4)	C22—C17—C18—C19	1.0 (4)
C1—C2—C7—C6	0.5 (4)	N2—C17—C18—C19	−178.7 (3)
C3—C2—C7—C6	−175.6 (3)	C17—C18—C19—C20	0.0 (5)
C1—C2—C7—C8	178.8 (3)	C18—C19—C20—C21	−0.4 (5)
C3—C2—C7—C8	2.8 (5)	C19—C20—C21—C22	−0.2 (5)
C6—C7—C8—C9	−0.6 (4)	C20—C21—C22—C17	1.1 (5)
C2—C7—C8—C9	−178.8 (3)	C18—C17—C22—C21	−1.5 (4)
C7—C8—C9—S1	40.3 (4)	N2—C17—C22—C21	178.2 (3)
C5—S1—C9—C8	−62.7 (3)		

### Hydrogen-bond geometry (Å, °)

**Table:**

*D*—H···*A*	*D*—H	H···*A*	*D*···*A*	*D*—H···*A*
C5—H5A···Cl1^i^	0.97	3.00	3.608 (3)	122.
C9—H9A···Cg1^ii^	0.97	2.84	3.778 (4)	163.

Symmetry codes: (i) −*x*+1, −*y*+1, −*z*+2; (ii) −*x*+2, −*y*+1, −*z*+1.
